# Exercise-based dysphagia rehabilitation for adults with oesophageal cancer: a systematic review

**DOI:** 10.1186/s12885-021-09155-y

**Published:** 2022-01-10

**Authors:** Anna Gillman, Michelle Hayes, Greg Sheaf, Margaret Walshe, John V. Reynolds, Julie Regan

**Affiliations:** 1 Department of Clinical Speech and Language Studies, Trinity College Dublin, 7-9 South Leinster Street, Dublin 2, Ireland; 2 Speech and Language Therapy Department, St James’ Hospital, James’ Street, Dublin 8, D08 NHY1 Ireland; 3 The Library of Trinity College Dublin, Dublin 2, Ireland; 4 Department of Surgery, St James’ Hospital, James’ Street, Dublin 8, D08 NHY1 Ireland

**Keywords:** Oesophageal cancer, Curative treatment, Dysphagia - swallowing rehabilitation, Swallow exercises

## Abstract

**Abstract:**

**Background:**

Dysphagia is prevalent in oesophageal cancer with significant clinical and psychosocial complications. The purpose of this study was i) to examine the impact of exercise-based dysphagia rehabilitation on clinical and quality of life outcomes in this population and ii) to identify key rehabilitation components that may inform future research in this area.

**Methods:**

Randomised control trials (RCT), non-RCTs, cohort studies and case series were included. 10 databases (CINAHL Complete, MEDLINE, EMBASE, Web of Science, CENTRAL, and ProQuest Dissertations and Theses, OpenGrey, PROSPERO, RIAN and SpeechBITE), 3 clinical trial registries, and relevant conference abstracts were searched in November 2020. Two independent authors assessed articles for eligibility before completing data extraction, quality assessment using ROBINS-I and Downs and Black Checklist, followed by descriptive data analysis. The primary outcomes included oral intake, respiratory status and quality of life. All comparable outcomes were combined and discussed throughout the manuscript as primary and secondary outcomes.

**Results:**

Three single centre non-randomised control studies involving 311 participants were included. A meta-analysis could not be completed due to study heterogeneity. SLT-led post-operative dysphagia intervention led to significantly earlier start to oral intake and reduced length of post-operative hospital stay. No studies found a reduction in aspiration pneumonia rates, and no studies included patient reported or quality of life outcomes. Of the reported secondary outcomes, swallow prehabilitation resulted in significantly improved swallow efficiency following oesophageal surgery compared to the control group, and rehabilitation following surgery resulted in significantly reduced vallecular and pyriform sinus residue. The three studies were found to have ‘serious’ to ‘critical’ risk of bias.

**Conclusions:**

This systematic review highlights a low-volume of low-quality evidence to support exercise-based dysphagia rehabilitation in adults undergoing surgery for oesophageal cancer. As dysphagia is a common symptom impacting quality of life throughout survivorship, findings will guide future research to determine if swallowing rehabilitation should be included in enhanced recovery after surgery (ERAS) programmes. This review is limited by the inclusion of non-randomised control trials and the reliance on Japanese interpretation which may have resulted in bias. The reviewed studies were all of weak design with limited data reported.

**Supplementary Information:**

The online version contains supplementary material available at 10.1186/s12885-021-09155-y.

## Background

Oesophageal cancer has an overall poor survival compared with many other malignancies. Recent reports indicate an approximate 5-year survival of between 15 and 25% [1–3]. However, 5-year survival has increased to approximately 50% amongst those who can be treated with curative intent [4]. Consequently, there is an emerging focus on enhanced recovery after surgery (ERAS) pathways to optimize clinical and health related quality of life outcomes amongst survivors (QOL) [5, 6]. For curative therapy, treatment usually involves surgery alone, either open or minimally invasive oesophagectomy (MIE). For locally advanced but curable disease, preoperative chemotherapy or a combination of chemotherapy and radiation therapy is increasingly a standard of care [7].

Dysphagia is a central symptom for the majority of patients with oesophageal cancer and the commonest presenting symptom [8, 9]. Prevalence of dysphagia is high in this population, with reports of dysphagia in 93% of patients with squamous cell carcinoma of the oesophagus, and in 79% of patients with adenocarcinoma of the oesophagus [10]. Most published research that reports on dysphagia in this population combines symptoms of dysphagia, without distinguishing between the different phases (for example oropharyngeal versus oesophageal dysphagia), therefore, it is not clear how prevalent each type of dysphagia is in isolation. Complications of a swallowing difficulty in oesophageal cancer include malnutrition, weight loss, muscle weakness and wasting (sarcopenia), and tube-feeding reliance [11–14].

There is little known about the prevalence and severity of oropharyngeal dysphagia in oesophageal cancer. Research that has been conducted to date has been limited and consists mostly of retrospective cohort studies. Despite this, early studies have shown that oropharyngeal dysphagia may exist following surgery and radiation therapy, as well as prior to any cancer treatment [15]. An important observation, albeit in a small study of 10 participants by Martin et al. [15], was that 9 out of 10 patients had mild oral-preparatory dysphagia and 100% of participants had at least mild oral and mild pharyngeal dysphagia prior to oesophageal cancer surgery. The characteristics most observed on videofluoroscopy (VFS) were impaired tongue movement, oral residue, hesitancy initiating the tongue stripping wave, impaired bolus formation, premature posterior spillage of the bolus from the oral cavity, delay in pharyngeal swallow initiation and post-swallow pharyngeal residue. VFS evaluation of swallowing also revealed altered hyoid trajectories in relation to the timing of its superior-most and anterior-most positions [15]. Post-surgery VFS studies have revealed that new-onset pharyngeal biomechanical impairments include reduced tongue pressure, reduced base of tongue to posterior pharyngeal wall approximation, delayed initiation of the pharyngeal swallow, reduced hyo-laryngeal elevation and excursion, reduced pharyngeal contraction, vocal fold immobility, and reduced maximum opening of the upper oesophageal sphincter during swallowing, resulting in symptoms such as overt and silent aspiration, and pharyngeal residue [9, 15–19].

Surgery for oesophageal cancer is associated with complications in between 50 and 60% of patients [20, 21]. Complications that may contribute to oropharyngeal swallowing deficits include anastomotic leaks, anastomotic scarring or inflammation, radiation-induced inflammation, fibrosis and strictures, endotracheal tube trauma, adhesion of the gastric tube to the trachea, and mechanical denervation and inflammation of key nerve pathways such as the vagus nerve, Ansa Cervicalis or the pharyngeal plexus [9, 15, 17, 19, 22–24]. A study by Mafune et al., 2019, reported recurrent laryngeal nerve paralysis in 65.6% of patients, as observed on laryngoscopy in 21 patients on post-operative day 1 [25]. Operation time greater than or equal to 6 h and vocal cord paralysis were found to be risk factors for subglottic aspiration with a high probability of occurrence (42.3%) if either risk factor was present [26]. The most likely mechanism impairments are insufficient vocal fold closure and impaired laryngeal sensation [27].

The most closely associated complication of surgery for oesophageal cancer is postoperative pulmonary complications (PPCs), in particular pneumonia, which occurs in approximately 25% of patients and is the most common cause of death [28, 29]. The presence of dysphagia postoperatively is an independent risk factor of pneumonia [30]. Berry et al., 2010, [30] found that 12% of patients had evidence of aspiration, hence approaches that minimise aspiration and aspiration pneumonia are clinically of great relevance to achieving optimum outcomes for these patients. Improving swallowing would be expected to reduce aspiration, reduce pneumonia and PPCs, and may impact on sarcopenia and malnutrition, treatment-related morbidity, hospital length of stay and readmissions [30–33].

### Health-related QOL complications of dysphagia in Oesophageal Cancer

The impact of a swallowing difficulty on the health-related QOL of a person with oesophageal cancer can also be devastating, leading to anxiety during meals and limiting participation in family mealtimes and social occasions [34]. Health-related QOL was found to be significantly impaired 10 years after both open oesophagectomy and MIE across many domains including dysphagia, reflux, eating difficulties, oesophageal pain, trouble swallowing saliva, choking, dry mouth and taste problems, with eating difficulties being one of the most outstanding problems [5, 35]. A study by Yuen et al., in 2019 [36], revealed that 29 survivors who were an average of 3.5 years post oesophagectomy, and who had no history of swallowing impairment, continued to present with a mild to moderate pharyngeal dysphagia on videofluoroscopy. Survivors have reported persistent swallowing difficulties 5 and 10 years after treatment, demonstrating that this is a chronic issue in need of addressing [5].

### Dysphagia rehabilitation in Oesophageal Cancer

There is limited research exploring swallowing rehabilitation in oesophageal cancer. In one recent systematic review, Kaneoka et al., 2018, [31] found four studies that evaluated dysphagia intervention in this population. Three of these four studies investigated the chin tuck postural strategy. This strategy compensates for a deficit during the act of swallowing, as opposed to rehabilitative exercises, which aim to induce long-term improvements to swallow function. Only one included study evaluated dysphagia rehabilitation in their review [37].

It is widely known that patients with head and neck cancer suffer significant short-term and long-term dysphagia, impacting upon their QOL and activities of daily living [38], and that swallow rehabilitation in head and neck cancer has improved QOL and dysphagia outcomes such as severity of aspiration and pharyngeal efficiency [39–45]. It is currently not known if and how swallow rehabilitation may improve swallowing, and if there are any contraindications for its use with patients with oesophageal cancer. Systematic review findings may guide clinical decision-making regarding dysphagia rehabilitation and inform future research in this area.

### Study aims

1. To determine the effectiveness of dysphagia rehabilitation in improving clinical outcomes (oral intake status, pneumonia and swallow) and health related QOL outcomes in adults with oesophageal cancer across time points.
2. To identify key elements of rehabilitation (delivery, dose, intensity, timing, adverse events and fidelity) which may inform future research of dysphagia rehabilitation in oesophageal cancer.

## Methods

### Registration

The guidelines from the Preferred Reporting Items for Systematic Reviews and Meta-Analysis (PRISMA) Statement [46] were adhered to. The protocol was registered on the PROSPERO database of prospectively registered systematic reviews (reference number CRD42020172029; https://www.crd.york.ac.uk/prospero/display_record.php?RecordID=172029).

### Search strategy

Six electronic databases (CINAHL Complete, MEDLINE, EMBASE, Web of Science, CENTRAL, and ProQuest Dissertations and Theses) were searched for eligible studies. One author (GS) designed and ran a systematic search across all six databases for the concepts “oesophageal cancer”, “dysphagia/deglutition” and “rehabilitation”, using controlled vocabulary and synonyms and related terms in the titles and abstracts, and then combined as appropriate (see Additional file 1 for an example of a database-specific search strategy). Four remaining databases (OpenGrey, PROSPERO, RIAN and SpeechBITE) were searched by the first author (AG) using the term ‘(o)esophageal cancer’ (see Additional file 1). All literature published since inception up until November 2020 was considered to ensure that a thorough search of the literature was completed. Publications from any country of origin and written in any language were deemed eligible, and were translated using online professional tools and a professional interpreter. Clinical trial registries (ClinicalTrials.gov, ISRCTN, WHO Trial Registry) were searched by the first author (AG). Reference lists of relevant studies and a manual search on Google Scholar were also completed by the first author (AG) to ensure literature saturation. In addition, the first author (AG) completed a manual search of relevant conference proceedings of the annual congresses of the Dysphagia Research Society, the European Society for Swallowing Disorders, the European Society for Diseases of the Oesophagus, and the International Society for Diseases of the Oesophagus from inception. Reference checks and citation tracking were conducted to ensure all relevant articles were retrieved for analysis. The reference manager software, EndNote, was used to manage references.

### Study selection

Inclusion and exclusion criteria were applied independently to the articles by 2 reviewers (AG, MH). All published and non-published peer-reviewed randomized control trials (RCTs), quasi-experimental designs, observational studies, conference presentations, abstracts and non-systematic reviews were included in the search criteria due to the limited number of publications in this area of research to date. Grey literature was searched to locate relevant non-published studies. The first author (AG) attempted to obtain further information on relevant studies reported in conference abstracts. Expert opinions, letters to editor, commentaries, editorials, and textbooks were excluded. Full texts were retrieved, and inclusion and exclusion criteria were re-applied independently to the articles by 2 reviewers (AG, MH).

### Eligibility criteria

#### Participants

Inclusion criteria for participants encompassed adults (≥ 18 years old) undergoing curative intent resection (oesophagectomy) +/− neoadjuvant therapy for oesophageal cancer, who had a swallowing disorder of any severity, and who participated in rehabilitative intervention to improve their swallowing disorders. An oesophagectomy is defined as a type of surgery whereby part of or all of the oesophagus is removed. Both methods of surgery (open or minimally invasive) were included. Participants of any gender and ethnicity with any stage and type of cancer at any location in the oesophagus were included. There was no restriction on settings. Participants were excluded if they presented with other conditions known to impact on swallowing (for example the presence/history of stroke, neurodegenerative disease, head and neck cancer) or if palliative treatment was being provided. Participants with gastric cancer were excluded.

### Interventions

Articles for inclusion were studies that investigated dysphagia rehabilitation in oesophageal cancer. All intensities and durations of rehabilitation were included. A clear definition of dysphagia rehabilitation exercises was created to ensure that results retrieved were not compounded by compensatory strategies. A dysphagia rehabilitative exercise was defined, for the purpose of this systematic review, as *an exercise which aims to create lasting functional change to the efficiency, strength, coordination and safety of an individual’s swallow by improving underlying physiological function, rather than compensating for a deficit in the moment*. This includes strength-based exercises, skill-based exercises and sensory-rehabilitative exercises. The main goal of strength training is the enlargement of the muscle fibres (hypertrophy) [47]. Skill-based training aims to modulate the cortex resulting in adaptive swallowing practices, such as increased precision and timing of swallowing [48]. The intent of sensory-based rehabilitation is to produce long-term changes in the organisation of sensory and motor areas of the cerebral cortex, as sensory input has been proven to alter the excitability of the motor cortex (cross-system plasticity) [49].

With respect to our review, dysphagia rehabilitation did not include compensatory interventions, which were defined as *strategies implemented at the time of swallowing that aim to temporarily compensate for the swallow dysfunction or compromised airway*, for example food/fluid texture and volume modification, head and neck postures, environmental/utensil/pacing modifications etc. Studies were excluded if their only focus was compensatory intervention. Studies that contained both rehabilitative exercises and compensatory strategies were included.

Dysphagia rehabilitation was considered for inclusion if it was prior to, during or at any time-point following participants’ surgical or neo-adjuvant treatment for oesophageal cancer.

### Comparison

Groups were considered for comparison of outcome measures if they received:

- No intervention
- Usual care that did not include rehabilitative exercises, for example compensatory strategies (as defined above).
- A placebo or control group (for example if patients received a sham intervention such as with Expiratory Muscle Strength Training when they breathe into a device with no resistance).
- The same or different dysphagia rehabilitation programs.

### Outcome measures

A comprehensive list and definition for all outcomes was prepared in line with the PRIMSA 2020 statement 10a [50]. Oral intake was considered to be the main outcome given its significant relevance to both clinicians and patients.

The three primary outcome measures for examination were:

1. Oral intake: Change in oral intake status, for example, feeding-tube reliance rated on the Functional Oral Intake Scale [51].
2. Respiratory Complications: Incidence of respiratory complications including pneumonia indicated by the presence of new/worsening chest x-ray or computed tomography (CT) results and defined by the Centre for Disease Control (CDC) [52] and the Standardized Endpoints for Perioperative Medicine (StEP) collaborative network [53].
3. Quality of Life: Change to swallow-related QOL scores on validated scales such as the MD Anderson Dysphagia Intervention Questionnaire [54] or SWAL-QOL (Swallow Quality of Life Questionnaire) [55].

The Secondary Outcomes were:

1. Change to patient-reported dysphagia ratings on validated scales, for example, the EAT-10 (Eating Assessment Tool- 10) [56].
2. Instrumental measures of swallowing including change in pharyngeal pressures as determined by pharyngeal high-resolution impedance manometry (PHRIM), change in timing of swallowing as determined by instrumental swallow evaluation and using a validated scale (such as the MBS Impairment Profile or the Dynamic Imaging Grade of Swallowing Toxicity-DIGEST), change in incidence, frequency or volume of laryngeal penetration/aspiration, and safety of swallowing, as determined by instrumental swallow evaluation (VFS or Fiber-Endoscopic Evaluation of Swallowing (FEES)), and a validated tool (such as the Penetration-Aspiration Scale (PAS) [57] or DIGEST [58]), change in efficiency/residue of swallowing as determined by instrumental swallow evaluation and using a validated scale (for example the MBS Impairment Profile [59] or DIGEST or PHRIM).
3. Time taken in days to return to oral intake.
4. Hospital length-of-stay in days.
5. Nutrition: Change to nutritional status such as using a validated tool for measuring weight loss, malnutrition, sarcopenia, cachexia, dehydration for example the MUST (Malnutrition Universal Screening Tool) [60].
6. Adverse events such as increased fatigue, deterioration of swallow function or patient discomfort.

All pre-intervention, peri-intervention and post-intervention (including long-term) outcomes measurements were recorded.

### Data extraction

Data was extracted independently by two authors (AG, MH) through a data extraction form specifically designed for the purpose of this study. If a study presented with any missing or unclear data, study authors were contacted in an attempt to resolve uncertainties. Disagreements were resolved by consensus and consultation with a third review author (JR). Four main domains were explored: participant characteristics; study characteristics; intervention characteristics; and outcomes of interest. Data regarding key intervention components such as delivery, intensity, timing, fidelity and adherence were collected using the TIDieR checklist (Template for Intervention Description and Replication) [61]. This instrument contains 12 items that describe a trial’s completeness of reporting of interventions. Yamato et al., 2018 [62], developed a TIDieR Summary Score for this tool, where elements are scored ‘0’ if something is not reported, ‘1’ if partially reported, and ‘2’ if adequately reported. Once data were extracted, they were merged into tabular forms on a Microsoft Excel spreadsheet.

### Data analysis

The authors intended to tabulate all information relevant to each outcome. The authors aimed to stratify data by patient characteristics such as tumour site, tumour type, surgical approach, site of anastomosis, partial resection/ total oesophagectomy in order to conduct thorough meta-analyses. If meta-analyses were not possible, the authors planned to conduct descriptive synthesis of the data, focusing on all primary and secondary outcomes. Information would be aggregated in a spreadsheet under appropriate outcome headings. Comparable outcomes would then be combined for discussion and included in tables.

### Assessment of methodological quality

Risk of bias assessment of the selected studies was completed independently by two authors using the ROBINS-I (Risk Of Bias In Non-randomised Studies - of Interventions) Tool [63]. An overall judgement about risk of bias was made regarding the entire study. The case series study is of a particularly weak study design and ‘No information’ was a common response to the signalling questions meaning that there is insufficient information to make a judgement on the risk of bias. As a result, the Downs & Black Checklist [64] was selected as an adjunct quality assessment tool given its suitability for assessing quality of randomised and non-randomised studies, and given that its questions are less specific in nature and allow more information to be elicited from studies of weaker designs. In this review, the scoring of item 27, that refers to the power of the study, has been modified as per previous research [65]. Total scores range from 0 to 28, with higher scores indicating a stronger methodological quality study. Hooper et al., 2008, suggest score ranges that correspond to levels of quality: Excellent (26–28), Good (20–25), Fair (15–19), and Poor (≤14) [66].

The GRADE approach (Grading of Recommendations, Assessment, Development and Evaluation) was conducted to determine the certainty of the systematic review as a body of evidence, as this may assist clinicians with the development of health care recommendations [67, 68].

## Results

### Study selection

**Fig. 1:**
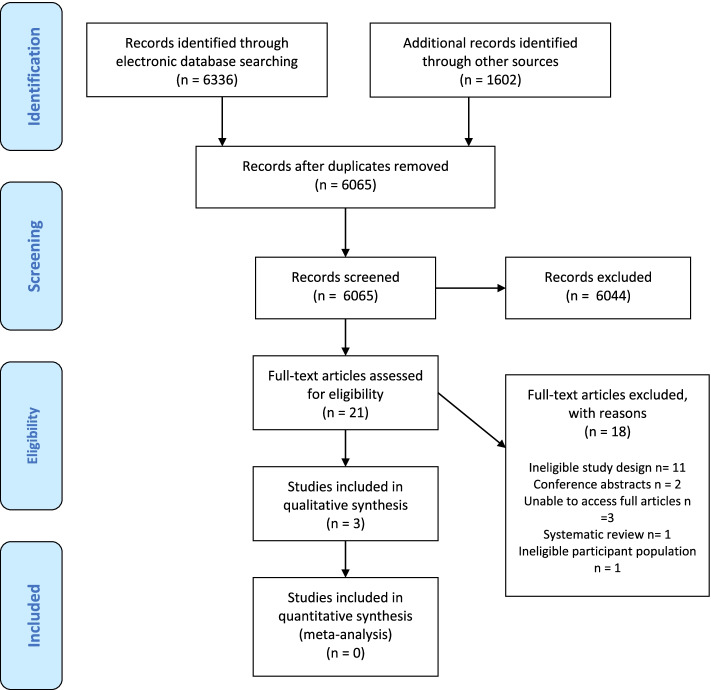
PRISMA 2009 Flow Diagram

Figure 1 presents the final study selection flowchart based on the systematic search. A total of 7938 articles were retrieved from the 10 databases and 3 Clinical Trial registries. Twenty-one full text articles were reviewed [AG & MH], eighteen of which were excluded mostly due to study design (such as review articles and observational studies). In line with the PRISMA 2020 statement, 16b [50], a list of the excluded full text articles, and reasons for their exclusion, can be reviewed in the Appendices (See Additional file 1). Three studies were included based on the inclusion and exclusion criteria outlined above. Included studies varied in methodological design (case control study, case series, retrospective case control study). A meta-analysis could not be completed due to the limited data in the studies and the heterogeneity across studies in terms of the participant characteristics, rehabilitation exercises implemented, the timing of the interventions, the measuring tools and the outcomes measures.

### Baseline characteristics

Three studies with a combined total of 311 participants who underwent oesophagectomy with thoracoscopy met inclusion criteria [37, 69, 70]. All three studies evaluated the impact of exercise-based dysphagia interventions on swallow related outcomes in adults with oesophageal cancer. Participants were enrolled over a minimum 15-year period up to December 2018. Each of these studies were completed in single centre settings in Japan. The authors were from different research teams at different hospitals. They focused on the short-term effects of peri-operative exercise-based dysphagia intervention on swallow-related outcomes. The case control study contained a control group, which comprised historically collected data from 14 participants who attended hospital from January 2012 to March 2013. The treatment group included 12 participants who attended hospital between April 2013 and September 2014 while a swallowing rehabilitation program was in place. There were 9 participants in the case series. In the third retrospective case control study, 167 participants who received dysphagia rehabilitation were compared to 109 patients who had previously received standard care. Demographic characteristics from the three included studies are outlined in Table 1.

**Table 1:** Demographic characteristics of included studies

Authors, year	Study Design	Study Setting	N	Age	Sex	Co-morbidities	Stage of cancer	Location of cancer	Type of cancer treatment	Complications post-surgery	Dysphagia Assessment for Outcomes
Okumura et al., 2016 [37]	Case Control Study (CCS)	Toyoma University Hospital, Japan	26	CG; Mean 65.9 +/− 9.7 yrs. TG: 68+/−5.1 yrs.	CG: 13/1 M:F TG: 12 M	NI	**TNM Classification (JES)** -I/II CG: 11 (78.6%) TG: 5 (41.7%) -III/IV CG: 3 (21.4%) TG: 7 (58.3%) **TNM Classification (UICC)** - I/II CG:12 (85.7%) TG: 8 (66.7%) -III/IV CG: 2 (14.3%) TG: 4 (33.3%)	Thoracic	“Oesophagectomy” 5 participants: neoadjuvant chemotherapy	CG: RLNP (n = 4) AP (n = 3) AL (n = 2) TG: RLNP(*n* = 2), AP (*n* = 3) AL (n = 2)	Non-validated: 1. Functional Outcomes Assessment Measure of Swallowing (FOAMS) Scale. 2 Measured relevant biomechanical positions and volumes on x-axis and y-axis plots from VFSS images
Tsubosa et al., 2005 [69]	Case Series (CS)	Shizuoka Cancer Hospital Rehabilitation Dept., Japan	9	Mean 57.8 +/− 9 yrs.	9 M	Cases 1: Hx of RTC; 2: old age; 3: Hx of stroke and abnormal shape of epiglottis; and 4: abnormal shape of epiglottis. The remaining 5 cases had no relevant co-morbidities that may affect swallowing	NI	Thoracic	“Oesophagectomy”	RLNP (*n* = 5. 1 of which was bilateral resulting in severe AP)	Non-validated: VFSS rating tool suggested by Logemann, 1998
Takatsu et al., 2020 [70]	Retrospective case control study	Aichi Cancer Centre Hospital, Japan	276	CG; median 68 (IQR 64–74) TG; 69 (IQR 62–73)	CG: 91/18 M:F TG: 142/25 M:F	NI	**TNM Classification (UICC)** -I/II CG: 49 (45%) TG: 56 (45%) -III/IV CG: 60 (55%) TG: 91 (55%)	Thoracic	CG: Neoadjuvant therapies: 87 (80%) Thoracoscopic oesophagectomy Open Oesophagectomy Cervical anastomosis TG: Neoadjuvant therapies: 133 (80%) Thoracoscopic oesophagectomy Open oesophagectomy Cervical anastomosis	CG: RLNP 22 (20%) Pneumonia 25 (23%) AL: 8 (7%) TG: RLNP 34 (20%) Pneumonia 39 (23%) AL 22 (13%)	1. Start of oral intake 2. Length of oral intake rehabilitation 3. Length of postoperative stay

Key: *Std Dev* Standard Deviation, *NI* No Information provided, *RLNP* Recurrent Laryngeal Nerve Palsy, *AP* Aspiration Pneumonia, *VFSS* Videofluoroscopy, *CG* control group, *TG* treatment group, *JES* Japan Esophageal Society, *UICC* Union for International Cancer Control, *Yrs* years, *AL* Anastomatic Leak, *Hx* history, *RTC* radiotherapy for tongue cancer

### Participant characteristics

Two-hundred and sixty-seven of 311 (86%) participants across studies were male. Within treatment groups, the average age (+/− standard deviation) was 68 (+/− 5.1) years in the case control study and 57.8 (+/− 9) in the case series, whereas median age was 69 years in the retrospective case control study. In the control groups [37, 70], 114/122 participants were male with an average and median age of 65.9 (+/− 9.7) years [37] and 68 years respectively [70]. Within the three studies, there were unmatched participant characteristics such as the severity of dysphagia experienced, co-morbidities, and the types, locations and stages of oesophageal cancer, surgical approach, site of anastomosis, partial resection/ total oesophagectomy. Four participants in the case series had medical histories which the study’s authors reported may have pre-disposed them to dysphagia, including history of radiotherapy for tongue cancer, ‘old age’, history of cerebral infarct and altered epiglottis shape respectively. Therefore, outcome results for these participants are not included in this review as per our exclusion criteria.

### Intervention characteristics

Intervention characteristics from the included studies are outlined in Table 2. None of the studies provided rationales for selection of the rehabilitation exercises based on swallow pathophysiology of this clinical population. Furthermore, no studies reported information to adequately address the second aim of this review, that is, regarding the mode, frequency, intensity, duration and dose of intervention.

**Table 2:** Intervention characteristics of included studies

Authors, year	Exercises with Rehabilitative Purpose	Other Exercises and/or Compensatory Strategies	Mode, Frequency, Intensity, Duration Dosage of Intervention	Timing of dysphagia rehabilitation in relation to start of cancer treatment	Duration of rehabilitation (Mean +/−Std dev)
Okumura et al., 2016 [37]	Pursed lip breathing, Tongue exercises, Shaker “head lift” exercises.	Cervical range of motion exercise Shoulder stretch Jaw opening Respiratory therapy Compensatory strategies: Modified food and fluids.	SLT & nurses in the surgical ward delivered initial verbal & written instruction. See Additional file 1 for instructions. Exercises × 5 a day at home and upon admission to the hospital, up until the day before surgery. Unclear if patient-led thereafter.	Prehab: Approximately 23+/− 9.2 days preoperatively Rehab: from the time oral intake was resumed after confirming the absence of anastomotic leakage post-surgery.	Prehab: 23+/−9.2 Days pre-surgery. Rehab: 26+/− 15 days post-surgery.
Tsubosa et al., 2005 [69]	Mendelsohn manoeuvre. Long lasting change may have also potentially occurred from the super-supraglottic swallow.	Oral care, Neck and shoulder exercises Oral exercises, Thermal tactile stimulation, Super-supraglottic swallow, Effortful breath hold. Compensatory strategies: Multiple swallows, chin down, Modified food and fluids.	Article states ‘Intensively’ however no definition or information provided. See Additional file 1 for information on exercises.	Post-operative- unknown precisely when.	9.7 +/− 6.9 days post-surgery. 5/9 participants required more than 1 round of rehabilitation.
Takatsu et al., 2020 [70]	Indirect training: Tongue exercises Shaker exercise Jaw opening Thermal-tactile stimulation Voice therapy	Direct training: Education provision Training while eating jelly: Position adjustment- chin down Effortful swallows, supraglottic swallow, adjusted bolus size supervised. Food and fluid intake increased based on patient progress.	No detail provided on duration, frequency or intensity of indirect or direct training.	Modified water swallow test (MWST) completed by SLT after routine CT on POD 5 or 6. Patients with intermediate or high aspiration risk based on MWST provided with indirect and, if possible, direct rehabilitation.	Not provided

Key: *Std Dev* Standard Deviation, *SLT* Speech and language therapist, *Prehab* Prehabilitation, *Rehab* Rehabilitation

### Rehabilitation results

Quantitative analysis of data in the three articles was not possible given the heterogeneity of studies (analysis conducted, interventions used, outcomes measured) and the small number of participants. Very few outcomes were reported by more than one study. Given the lack of core outcome sets for this type of research, and the few existing studies, there were insufficient and inadequate data that could be pooled together to conduct quantitative meta-analysis. Therefore, descriptive synthesis of the data was conducted focusing on the primary and secondary outcomes. All information provided was initially aggregated in a spreadsheet under an appropriate outcome heading. Comparable outcomes were combined for discussion and included in tables. All outcome results are discussed below. The key results are outlined in Table 3. Results are presented across outcome categories below.

**Table 3:** Summary of Reported Primary and Secondary Outcomes

Authors, year	Oral intake	Respiratory	Instrumental Swallow Outcomes			Nutrition	Time to return to oral intake: Days (average +/− SD)	LOHS after surgery: Days (average ± SD)	Quality Assess-ment	Tidier Check-list
			Penetration/ Aspiration	Pharyngeal Residue	Biomechanical Change to Swallow					
Okumura et al., 2016 [37]	FOAMS scores suggest primary mode of intake prior to and following surgery for all participants was oral means of nutrition.	AP: CG = 3pts (21.4%) TG = 3pts (25%) p = 0.83 Rehospitalisation for pneumonia within 3 months after surgery: CG =3 (21.4%), TG = 0.	Not reported h/e FOAM scores of 4,5,6 post- surgery and post- rehab suggest compensatory strategies needed, which may indicate risk of pen/asp. Number of participants with these scores not provided.	4 participants had pyriform sinus residue prior to prehabilitation; the volume decreased significantly following prehabilitation, with a *p* value of 0.047 Between start of rehab post-surgery and post rehab: volume of laryngeal vestibule and PS residue decreased significantly (*p* values of 0.031 and 0.027, respectively)	From prior to prehab to post prehab, and from post-surgery to post-rehabilitation: the TG’s maximum superior excursion of hyoid bone increased significantly during swallowing with p values of 0.03 and 0.046 respectively. No significant difference between the maximum anterior excursion of the hyoid bone or the anteroposterior diameters of the UES	Not reported. Body weight change 3 months after surgery (%, average +/− SD) was CG: 90.6% +/− 5.5 TG: 91.4% +/− 5.8 (p value = 0.36)	CG: 9.6+/− 5.3 TG: 11+/− 5.5 (*P* = 0.32)	CG: 32.4 ± 12.2 TG: 36.1 ± 10.7 (*p* = 0.22)	ROBINS-I: Serious Downs & Black: 13 (poor)	9
Tsubosa et al., 2005 [69]	Data provided not clear.	1 participant developed ‘severe‘AP. Other severities of AP not mentioned. No post-discharge AP.	Data available for 2 participants only: in 1 participant mild aspiration improved to normal. In 2nd participant, severe penetration and aspiration did not improve, but severe silent aspiration improved to normal.	Limited f/u data available h/e no improvement noted in the 1 participant with mild vallecular and PS residue	NI	NI	One participant: diet recovered to ‘independence’ on the 6th day. Otherwise, unclear when oral intake recommenced.	25.3 days for 8 participants. 96 days for remaining participant. No further detail given.	ROBINS-I: Critical Downs & Black: 1 (poor)	5
Takatsu et al., 2020 [70]	NI	NI	NI	NI	NI	NI	Start of oral intake significantly earlier in treatment group TG: 8 days (6–13) CG: 11 days (8–14)	CG: 22 days (17–27) TG: 19 days (15–27.5)	ROBINS-I: Serious Downs & Black: 15 (fair)	10

Key: *Pen/Asp* penetration/ aspiration, *QOL* Quality of Life, *LOSH* Length of stay in hospital, *NI* No information, *RLNP* Recurrent Laryngeal nerve paralysis, *AP* aspiration pneumonia, *AL* Anastomatic leak, *CG* control group, *TG* treatment group, *PS* pyrifom sinus, *UES* Upper oesophageal sphincter, *Pts* participants, *H/e* however, *F/u* Follow up

### Primary outcomes

#### Extent of oral intake

In one study [37], participants underwent dysphagia prehabilitation prior to surgery, as well as dysphagia rehabilitation following surgery. Both the treatment and control groups scored 7 out of 7 on the Functional Outcomes Assessment Measure of Swallowing (FOAMS) Scale prior to prehabilitation and following prehabilitation, indicating efficient and functional swallowing. The FOAMS score following surgery and prior to rehabilitation was higher for the treatment group than the control group, with average (+/− standard deviation) scores of 5.6 (+/1.2) and 4.7 (+/− 1.4) respectively, with a *p* value of 0.054, where 7 indicates functional, safe and efficient swallowing, and 0 indicates a profound swallow impairment necessitating only non-oral means of nutrition.

FOAMS scores at discharge were significantly worse than prior to prehabilitation for the control and treatment groups with average (+/− standard deviation) scores of 5.5 (+/− 1.3) and 6.3 (+/− 0.8) respectively (*p* value ≤0.01 for both groups). Perioperative swallow rehabilitation resulted in significantly higher swallow function scores for the treatment group discharge compared to the control group (p value = 0.049).

### Respiratory complications

One study evaluated respiratory complications and found no significant difference in aspiration pneumonia rates between a dysphagia rehabilitation treatment group (25%) and a control group (21.4%) (*p* = 0.83) [37]. In this same study, there was a non-significant reduction in rehospitalisation for pneumonia within three months of surgery in the treatment group (*n* = 0) compared to controls (*n* = 3). Of note, a clear definition of aspiration pneumonia was not provided in this study [37].

 *None of the studies reported on QOL outcomes*.   

 **Secondary Outcomes:**

#### Instrumental measures of swallowing

One of three included studies evaluated swallow biomechanics using VFS in the treatment group only [37]. Within this treatment group, the VFS examination took place pre-prehabilitation, pre-operatively and an average of 11 days (SD 5.5) post-esophagectomy. A significant increase in maximum superior excursion of the hyoid bone during swallowing was observed with intervention (*p* = 0.046), whereas anterior hyoid movement and anterior-posterior diameters of upper oesophageal sphincter (UOS) opening did not change. Of note, the methods used to measure hyoid excursion and UOS opening during VFS were not validated [37].

One study evaluated a change in pharyngeal residue with dysphagia intervention [37]. In this study, residue was rated based on the VFS evaluation by measuring volume (height x weight) of pharyngeal residue after the initial swallow (37). Based on VFS, a significant decrease in pharyngeal residue was found following prehabilitation in a subgroup of patients with pyriform residue at baseline (*p* = 0.047) (*n* = 4). In another study [69], no improvement was noted in one participant with residue in the valleculae and pyriform sinuses although a validated rating scale was not used.

Change in aspiration status was only reported on two participants in one study [68]. In these two cases, “mild” aspiration improved to “normal”, and “severe” silent aspiration improved to “normal”. A validated rating scale to measure aspiration was not used.

### Length of hospital stay

Two studies evaluated effects of dysphagia intervention on post-operative length of hospital stay (PLOHS) and they had conflicting results [37, 70]. While a more recent post-operative dysphagia intervention combining indirect and direct rehabilitation significantly shortened PLOHS from 22 (17–27) to 19 days (15–27.5) (*p* = 0.001) [69], PLOHS was increased from 32 (+/− 12) to 36 (+/− 11) days in a smaller, combined pre- and post-operative rehabilitation study, although this increase was not significant (*p* = 0.22) [37] (Table 3).

#### Time to return to oral intake

Two of three studies measured time to return to oral intake with conflicting results. One small study found no reduction in return to oral intake with rehabilitation (9.6 v 11 days; *p* = 0.32) [36], whereas a more recent, larger study found a significantly shorter return to oral intake in the dysphagia treatment group compared to the control group (11 v 8 days; *p* = 0.009) [70].

### Nutrition

One study found no significant difference in weight change between dysphagia rehabilitation treatment (91.4% +/− 5.8) and control (90.6% +/− 5.5) groups three months post-surgery (*p* = 0.36) [37]. No other nutritional measures were used across the included studies.

 *None of the studies noted patient-reported dysphagia outcomes or adverse events in hospital.*

### Quality assessment

The quality of all three studies was limited by numerous factors such as measuring outcomes using non-standardised rating tools, not documenting inclusion and exclusion criteria, implementing rehabilitation exercises without clear rationale based on swallow pathophysiology, and not considering or accounting for the impact of other treatments that participants may have been receiving at the same time. Inadequate analyses were conducted in the case control study and the case series, for example, neither study reported confidence intervals, standard error of means or estimates of random variability.

The case series was particularly weak. Participants were discharged if they did not show overt signs of aspiration with a whole meal of porridge and fluids; an instrumental assessment was not completed meaning that participants may have been discharged with subclinical aspiration, and there was no report of any further monitoring of their respiratory statuses. As a result of not conducting a VFS at the end of rehabilitation, there was no follow up data available for 4/9 participants. Only those who required a further round of rehabilitation had one additional VFS prior to starting the second round of rehabilitation. There was also a lack of clarity regarding definitions such as ‘mild aspiration’ and ‘severe aspiration’. Finally, authors of the case series reported that 8 participants did not achieve ‘severe’ aspiration pneumonia, however, other severities of pneumonia are not reported on, nor the method for measuring this outcome.

Based on the ROBINS-1, the case control study [37] and retrospective case control study [70] were found to have a serious risk of bias in one domain ‘Bias of Confounding’, and low risk of bias or ‘No information’ across all other domains. As the lowest domain score is considered to be the overall risk of bias for the study, these studies were deemed to have a ‘serious’ risk of bias. With regard to the case series, two domains scored ‘Low’; ‘Bias in classification of intervention’ and ‘Bias due to deviations from intended interventions’, however ‘Bias of Confounding’ was found to be at a ‘critical’ risk of bias. Therefore, this study is given an overall rating of a ‘critical’ risk of bias. According to the Downs and Black checklist, the retrospective case control study was deemed to be fair quality [70] and the other two studies were low quality [37, 69] (please refer to Appendices 5 and 6 for more information on the ROBINS-I and the Downs and Black Checklists respectively). The TIDieR checklist demonstrated relatively low scores regarding completeness of reporting of the interventions (checklist score range 5–10). While the retrospective case-control study [70] scored 10/24, the case control study scored 9/24 and the case series scored 5/24 (see Additional file 1).

## Discussion

### Main findings

This systematic review found only three eligible studies, including a total of 311 participants, that examined the effects of exercise-based dysphagia rehabilitation in adults with oesophageal cancer. It was not possible to complete a meta-analysis because of the lack of comparable data that could be pooled together. These studies provided limited evidence to support dysphagia rehabilitation in this clinical population. The limited evidence was restricted to clinical and functional outcomes including time to oral intake and length of hospital stay. None of the included studies investigated the impact of dysphagia intervention on quality of life or patient reported outcome measures, and there was very limited data on changes to swallow biomechanics and aspiration status. Furthermore, no data was obtained relating to the second aim of this study, for example, delivery, dose, intensity, timing, adverse events and fidelity which may have assisted with directing future research of dysphagia rehabilitation in oesophageal cancer. Finally, included studies focused on prehabilitation and post-surgical rehabilitation before hospital discharge, whereas no studies were found which evaluated the long-term benefit of dysphagia intervention post discharge in the community. While identification of these gaps in the evidence base is of concern, these issues can be addressed within future research in this area.

VFS results in the case series confirmed the presence of oral and pharyngeal dysphagia in participants who received an oesophagectomy and otherwise had no relevant medical history [69]. A small number of cases were followed up (*n* = 2) following post-surgical rehabilitation and authors reported improved laryngeal elevation, reduced aspiration and reduced silent aspiration across these participants. Perioperative rehabilitation in the case series [37] resulted in significantly better swallow function scores for the treatment group compared to the control group at the time of discharge, indicating that swallow function is improved by perioperative dysphagia rehabilitation. Interestingly, the treatment group was found to have better swallow efficiency following surgery than the control group indicating that exercises conducted during prehabilitation may help to minimise the impact of surgery on swallowing. Further high-quality research investigating the benefits of prehabilitation is warranted. Despite these gains, the case control study did not find significant improvements to respiratory status, time to return to oral intake or hospital length of stay following surgery.

The authors of the case series suggested that there is a high risk of severe aspiration in participants with bilateral recurrent laryngeal nerve paralysis, but not with unilateral palsy. The retrospective case study found that 20.3% of participants experienced recurrent laryngeal nerve paralysis (RLNP) as diagnosed by head and neck surgeons via fiberoscopy. 66.7% of these patients were found to have moderate or low risk of aspiration on videofluoroscopy at least day 5 days post-operatively.

The impact of presbyphagia, meaning normal healthy age-related swallow changes, must be taken into account when considering the swallowing presentation of this cohort as signs of presbyphagia do not constitute a swallowing impairment and must not be considered as oropharyngeal manifestations in people with oesophageal cancer. For example, 39% of healthy adults with a mean age of 73 presented with pharyngeal residue on FEES, in a study by de Lima et al. in 2018 [71], however, 15% of oesophageal cancer participants in the case control study [37] experienced a significant increase in residue following oesophagectomy compared to pre-surgery (*p* = 0.003 and *p* = 0.0031 for laryngeal and pyriform sinus residue respectively)*.* Any potential impact of age related swallow changes may be relevant when considering the two main cancer types; in particular individuals with adenocarcinoma, as the global incidence rate of oesophageal cancer is highest in adults over 70 years of age [72], with incidence of oesophageal adenocarcinoma in the US peaking at 80–84 years for men and 85 years + for women [73]. The global median age of squamous cell carcinoma has been reported to occur at 67.5 years [74]. It is noted that the mean age across the three included studies is approximately 65 years, with no details provided on the types of cancer that participants presented with.

### Existing research

Despite the significant impact of dysphagia throughout the oesophageal cancer journey, both from a clinical perspective and on an individual’s QOL, little is known about the benefit of dysphagia rehabilitation to optimise oropharyngeal swallowing in this population. A previous systematic review completed by Kaneoka et al., 2018 [31], focused on four questions, one of which examined the efficacy of rehabilitative interventions for oropharyngeal dysphagia in oesophageal cancer. Their search, which was conducted in August 2017, was limited to 5 databases and only included studies which used VFS or FEES, and which contained more than 5 participants. Three of the 4 studies included in their review focused on compensatory strategies to temporarily optimise swallowing, and 1 focused on rehabilitative exercises. Consequently, their review differs from our systematic review, which consisted of a broad literature search of 10 databases, 3 clinical trial registries and proceedings from 4 conferences to ensure literature saturation and included studies that did or did not use instrumental assessment. We focused specifically on exercise-based dysphagia rehabilitation by developing a definition that would differentiate rehabilitative exercises from compensatory strategies to focus only on long-lasting functional change. Our results retrieved an additional case series and retrospective case control study.

Multi-disciplinary management of oesophageal cancer is strongly recommended to maximise recovery, including the improvement of clinical and QOL outcomes [7, 75–78]. In addition to medical and surgical interventions, rehabilitation can be provided to patients prior to, during or following oesophageal cancer treatment. Cancer prehabilitation involves the delivery of rehabilitation between diagnosis and acute treatment to reduce post-operative morbidity and improve functional recovery, and it has been found to improve clinical outcomes in oesophageal cancer [78]. Bolger et al., 2019 [79] completed a systematic review on prehabilitation and rehabilitation in oesophagogastric malignancies and found that a preoperative exercise programme led to a reduction in perioperative morbidity, most notably pulmonary complications. These findings are significant given that a proportion of patients do not progress to curative treatment because of physiological and nutritional deterioration [80].

Recent advances in dysphagia research include the development of validated rating scales, improved understanding of optimum intervention dosages, the development of new interventions guided by underlying swallow pathophysiology and better awareness of adherence promotion and goal-oriented treatment. As a result of this and improved research quality, the evidence base for dysphagia rehabilitation is emerging across clinical populations including head and neck cancer [38–45] where a need for further high quality research appears to be under way in a proposed randomised control trial by Martino et al. [81]. There is a strong rationale for exercise-based dysphagia rehabilitation in oesophageal cancer to optimise clinical and quality of life outcomes. However, robust research is required to identify the nature of this intervention to guide clinical practice.

### Study limitations

This systematic review has a number of limitations that warrant consideration. Firstly, given the relatively new field of dysphagia rehabilitation, all study designs were eligible for inclusion in this review. As a result, included studies presented with weak study designs and limited data. The inclusion of non RCT studies in a systematic review may impact upon confidence in interpreting findings, because of potential biases, which the authors have attempted to minimise through implementing a rigorous, methodological approach when conducting this review. A meta-analysis was not possible due to the heterogeneity of these non-randomised control trials, particularly in terms of evaluation methods, outcome data, timing and type of intervention. Further information regarding the differences between the studies is listed in Tables 1 and 2, and expanded upon under Quality Assessment. The study authors all utilised non-validated outcomes meaning that the results outlined may be unreliable. The lack of information on the intensity, frequency and duration of intervention was alarming, and as a result, it was not possible to address the second aim of this review. In addition to this, the case series did not define its exercises. Consequently, it was not possible to confirm with certainty which exercises met our definition of rehabilitative exercises. Many of the outcomes anticipated by authors of this review were not reported on in either study including quality of life measures, self-reported dysphagia outcomes, and adverse events. The most recent study included in this review, the retrospective case control study [70], was of the highest methodological quality which is promising. Nonetheless, non-blinding of raters and limited information on co-morbidities and intervention dosage limited the rigour of this study. As a result, the quality of this body of evidence as a whole is deemed to be of ‘low quality’, as per the GRADE approach [67, 68]. This may of course change when more evidence of higher quality is published.

Secondly, this systematic review included articles in any language. Dutch, Japanese and German articles were excluded at the title/abstract screening stage using online translation tools and with assistance from a professional Japanese interpreter. The case series full article was professionally translated from Japanese characters to English. As a result of using Japanese interpreters and online translation tools, some information may have been misunderstood, which potentially could have contributed towards the Reporting Bias perceived in the case series. Finally, this systematic review excluded letters to editors which may potentially have included useful information about trials, however, none were retrieved during the screening. Despite these limitations, an important purpose of systematic reviews is to demonstrate when evidence is lacking in a particular area to guide future research [82] as observed in previous studies [83]. According to the Cochrane Collaboration, although a minimum of two studies is required to complete a meta-analysis, there is no minimum number necessary to complete a systematic review, as long as relevant studies have been retrieved [84–87].

### Implications for policy, practice and future research (using EPICOT) [88]

This review highlights the need for randomised controlled trials to evaluate the effectiveness of dysphagia rehabilitation in improving swallowing function and safety in adults with a diagnosis of oesophageal cancer. Important outcomes to be measured include extent of oral intake, incidence rates of aspiration pneumonia, swallow-related quality of life, instrumental measures of swallowing including aspiration, patient reported dysphagia outcomes, time to return to oral intake, length of time in hospital, nutrition, and adverse events. Research leading to the development of core outcomes sets to be used across studies would help to provide comparable data for the assessment of efficacy. Clear data should be provided on participant characteristics including tumour histology, tumour location, surgical approach, partial/total oesophagectomy, anastomosis site, co-morbidities, sex, age, ethnic group, and inclusion or exclusion criteria. The importance of rehabilitation dosage and frequency, adherence promotion, biofeedback and goal-oriented treatment is recognised in dysphagia research and should all be integrated into intervention design. Adverse events should also be commented on given their relevance for both clinicians and patients, and the potential to decrease patient adherence to rehabilitation programmes.

## Conclusion

Despite the prevalence and impact of dysphagia in oesophageal cancer, no systematic review has previously attempted to summarise the evidence for exercise-based dysphagia rehabilitation in patients receiving curative treatment for oesophageal cancer. This systematic review included three studies and no meta-analysis was possible due to study heterogeneity. It revealed that there is very limited low-quality evidence that dysphagia rehabilitation may result in functional swallowing improvements, faster return to oral intake and reduced length of hospital stay. However, no evidence regarding reduction in aspiration rates or aspiration pneumonia and no patient reported outcomes were found. As survivorship for this population is increasing, the findings from this review will guide the design of future high-quality research in this area.

## Supplementary Information

**Additional file 1.**

## Data Availability

The datasets used and/or analysed during the current study are available from the corresponding author on reasonable request.

## Abbreviations

CDC: Centre for Disease Control
DIGEST: Dynamic Imaging Grade of Swallowing Toxicity
EAT-10: Eating Assessment Tool- 10
ERAS: Enhanced recovery after surgery
FEES: Fiber-Endoscopic Evaluation of Swallowing
FOAMS: Functional Outcomes Assessment Measure of Swallowing
GRADE: Grading of Recommendations, Assessment, Development and Evaluation
MBS: Modified barium swallow
MDADI: MD Anderson Dysphagia Intervention Questionnaire
MIE: Minimally invasive oesophagectomy
MUST: Malnutrition Universal Screening Tool
PAS: Penetration-Aspiration Scale
PHRIM: Pharyngeal high-resolution impedance manometry
POLHS: Post-operative length of hospital stay
PPC: Postoperative pulmonary complications
PRISMA: Preferred Reporting Items for Systematic Reviews and Meta-Analysis
QOL: Quality of life
RCT: Randomised control trials
RLNP: Recurrent laryngeal nerve paralysis
ROBINS-I: Risk Of Bias In Non-randomised Studies - of Interventions
StEP: Standardized Endpoints for Perioperative Medicine
SWAL-QOL: Swallow Quality of Life Questionnaire
TIDieR: Template for Intervention Description and Replication
UOS: Upper oesophageal sphincter
VFS: Videofluoroscopy
