# Supplementary material for: Exercise-based dysphagia rehabilitation for adults with oesophageal cancer: a systematic review
Source: BMC Cancer. 2022 Jan 10;22:53. doi: 10.1186/s12885-021-09155-y (PMC8751332; doi:10.1186/s12885-021-09155-y)
Supplement: Supplementary file 1 — Additional file 1. [file 12885_2021_9155_MOESM1_ESM.docx]

**Appendices**

**Appendix 1:**

**Search strategy for MEDLINE**

1. (MH "Deglutition") OR (MH "Deglutition Disorders+") OR TI ( dysphagi* OR deglutition* OR swallow* OR ((feeding OR eating OR drinking) N3 disorder*) ) OR AB ( dysphagi* OR deglutition* OR swallow* OR ((feeding OR eating OR drinking) N3 disorder*) )
2. (MH "Gastrointestinal Neoplasms+") OR TI ( (esophag* OR oesophag* OR gastrointestinal OR "gastro-intestinal") N3 (cancer* OR neoplasm* OR carcinoma* OR tumor* OR tumour*) ) OR AB ( (esophag* OR oesophag* OR gastrointestinal OR "gastro-intestinal") N3 (cancer* OR neoplasm* OR carcinoma* OR tumor* OR tumour*) )
3. (MH "Exercise+") OR (MH "Rehabilitation+") OR TI ( Intervention* OR exerci* OR rehabilit* OR prehabilitat* OR "pre-habilitat*" OR treat* OR therap* ) OR AB ( Intervention* OR exerci* OR rehabilit* OR prehabilitat* OR "pre-habilitat*" OR treat* OR therap* )

Search is 1 AND 2 AND 3. No limits as to language or date in search.

**Appendix 2:**

**Search strategy for Open Grey, PROSPERO, RIAN and Speech Bite**

- Esophageal cancer OR
- Oesophageal cancer

**Appendix 3:**

**Reasons for excluding full texts with references**

**Ineligible study design**

1. Hirose, Chikako; Takeuchi, Hiroya; Ishikawa, Aiko; Kawakubo, Hirofumi; Tsuji, Tetsuya; Kitagawa, Yuko [Perioperative Rehabilitation for Patients with Esophageal Cancer] Kyobu geka. The Japanese journal of thoracic surgery 2016;69(1):53-58 Japan Nankodo 2016
2. Navaneethan, Udayakumar; Eubanks, Steve Approach to patients with esophageal DysphagiaThe Surgical clinics of North America 2015;95(3):483-489 United States W.B. Saunders 2015
3. Romanelli, Angela; Bozzone, Anna; Magrone, Giovanni; Di Lello, Simona; Pascarella, Biancalisa; Sterzi, Silvia Dysphagia following treatment for esophageal cancer: rehabilitation strategies Rays 2006;31(1):53-55 Italy Masson Italia Periodici 2006
4. Joseph, Ranjit; Laks, Shachar; Meyers, Michael; McRee, Autumn J. Multidisciplinary Approach to the Management of Esophageal Malignancies World journal of surgery 2017;41(7):1726-1733 United States Springer International 2017
5. Evangelista, Lisa M.; Coyle, James L. Considerations in Dysphagia Management Following Esophagectomy Perspectives of the ASHA Special Interest Groups 2016;1(13):169-176 Rockville, Maryland American Speech-Language-Hearing Association 2016
6. Vowinkel, T.; Tübergen, D. Dysphagia treatment in advanced esophageal cancer: Commentary Onkologe 2005;11(2):212-213 2005
7. Gimigliano, Raffaele; Bertella, Marianna; Gimigliano, Francesca; Iolascon, Giovanni Rehabilitation in esophageal cancer Rays 2005;30(4):295-298 Italy Masson Italia Periodici 2005
8. Bax, H. R. [New therapeutic possibilities in esophageal cancer] Geneeskundige gids 1952;30(14):301-307 Netherlands Moormans Periodieke 1952
9. Ansari, A. Carcinoma of the esophagus: diagnosis and treatment Geriatrics 1971;26(5):98-104 United States Advanstar Communications 1971
10. Oliveira, M. A.; Holscher, A. H.; Feussner, H.; Daschner, C.; Hannig, C.; Siewert, J. R.; Pinotti, H. W.; Nabeya, K.; Hanaoka, T.; Nogami, H. Swallowing Function before and after Esophagectomy for Treatment of Esophageal Cancer Recent Advances in Diseases of the Esophagus 1993;():746-751 1993
11. Griffin, S. M.; Robertson, C. S. Non-surgical treatment of cancer of the oesophagus The British journal of surgery 1993;80(4):412-413 England Wiley 1993

**Conference abstracts**

- Sadanaga, N, Honbo, T., Matsuura, Evaluation and Training of Swallowing for Postoperative Esophageal Cancer Patients. Diseases of the Esophagaus (2016) 29, 3A-162A DOI 10.1111/dote.12528
- Yagawa, Y., Narumiya K., Ohta, M., Kudo, K., Shirai Y., Ide, H., Osugi, H., Yamamoto, M. Multimodal Approach for Postoperative Pneumonia after Esophagectomy for Esophageal Cancer. Diseases of the Esophagaus (2016) 29, 3A-162A DOI 10.1111/dote.12528

**Unable to access full articles**

1. Mármol, M.; Maurel, J. Esophageal cancer: Multimodal treatment Gastroenterologia y Hepatologia Continuada 2007;6(2):64-67 2007
2. Minsky, Bruce D. Choosing the correct modality for the treatment of dysphagia in the patient with esophageal cancer The journal of supportive oncology 2006;4(8):377 United States Frontline Medical Communications 2006
3. Pradhan, S. A.; Rajpal, R. M. Modified treatment plan to restore early swallowing in oesophageal cancer Indian Journal of Cancer 1983;20(2):113-115 India Medknow Publications 1983

**Systematic review**

1. Kaneoka, A.; Yang, S.; Inokuchi, H.; Ueha, R.; Yamashita, H.; Nito, T.; Seto, Y.; Haga, N. Presentation of oropharyngeal dysphagia and rehabilitative intervention following esophagectomy: a systematic review
2. Diseases of the esophagus : official journal of the international society for diseases of the esophagus 2018;31(8):United States Oxford University Press 2018

**Ineligible participant population**

1. Borrett, Kevin INTERVENTIONS FOR DYSPHAGIA IN ESOPHAGEAL CANCER Gastroenterology nursing : the official journal of the Society of Gastroenterology Nurses and Associates 2018;41(1):73-75 United States Lippincott Williams & Wilkins 2018

**Appendix 4:**

**Exercise instructions for the Case control study ([37]**

| (1) **Pursed-lip breathing**  Sit up straight, relax your neck and shoulder muscles  Breathe in (inhale) slowly through your nose for two counts, keeping  your mouth closed  Pucker or “purse” your lips as if you were going to whistle or gently  flicker the flame of a candle  Breathe out (exhale) slowly and gently through your pursed lips  while counting to four  (2) C**ervical range of motion exercise**  Head tilts, forward and back  Gently bow your head and try to touch your chin to your chest  Hold for 10 s  Raise your chin back to the starting position  Tilt your head back as far as possible so you are looking up at the  ceiling  Hold for 10 s  Return your head to the starting position  Head tilts, side to side  Tilt your head to the side, bringing your ear toward your shoulder  Hold for 10 s  Return your head to the starting position  Head turns  Turn your head to look over your shoulder  Tilt your chin down and try to touch it to your shoulder  Hold for 10 s  Return your head to the starting position  (3) **Shoulder stretch**  Reach up over your head with both arms  Squat putting the arms around your knees with breathing out and  put your head down to look at your tiptoes  Hold for 10 s  Return to the starting position  (4) **Jaw opening**  Open your jaw as wide as you can until you feel a stretching, but no  pain  Hold this maximum open position for 10–30 s  Relax and close your mouth  Repeat this open position 5 times  (5) **Tongue exercises**  Tongue extension  Poke your tongue out between your lips  Stick out your tongue as far as you can  Hold the tongue steady and straight for 10–30 s  Relax and then repeat 5 times.  Tongue retraction  Retract your tongue, touching the back of your tongue to the roof  of your mouth  Hold for 1–3 s  Relax and then repeat 5 times  Tongue tip up  Put the tip of your tongue behind your top teeth  Open your mouth as wide as possible maintaining tongue contact  Hold for 3–5 s  Relax and then repeat 5 times  Tongue tip down  Table 1 continued  Put the tip of your tongue behind your bottom teeth  Open your mouth as wide as possible maintaining tongue contact  Hold for 3–5 s  Relax and then repeat 5 times  (6) **Shaker exercises**  Sustained hold  Lift your head to look at your toes  Keep your shoulders flat on the floor/bed  Hold this position for 30 s  Release  Repeat 3 times and rest for 1 min between repetitions  Lift and lower (same starting position as for sustained hold)  Lift your head and look at your toes  Let your head go back down with control  Repeat 30 times  Rest in between as needed |
| --- |

**Appendix 5:**

**Therapy procedure and corresponding purpose** **for the Case Series** [69]


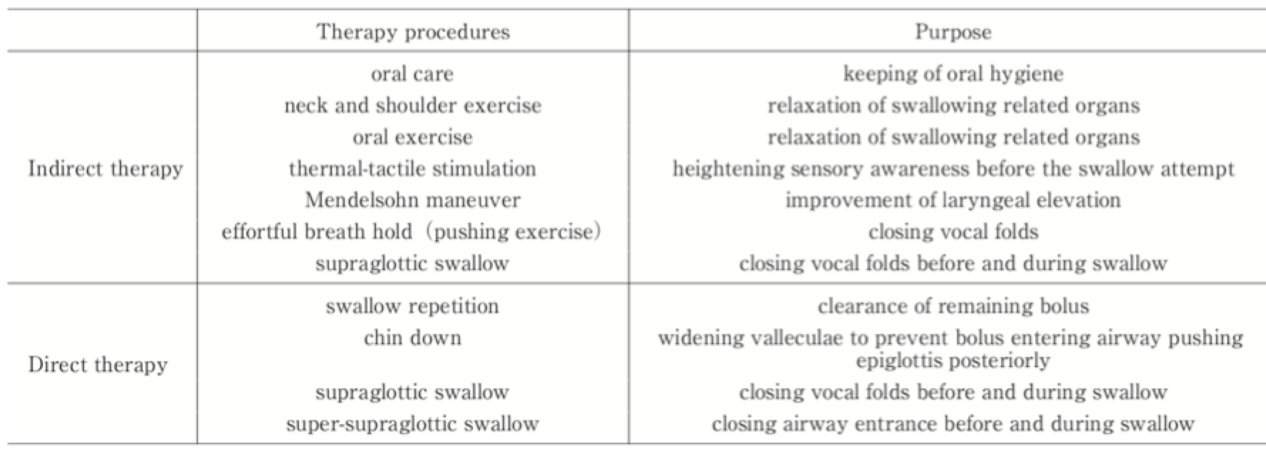


**Appendix 6:**

**Risk Of Bias ROBINS- I Results**

Case Control Study [37]

|  | FOAMS | Asp  Pneumonia | Duration b/w op and oral intake | Dur b/w oral intake and d/c | Dur b/w sx and d/c | Pneum within 3 mo | Pharyngeal clearance | Max hyoid superior | Max hyoid anterior | UES |
| --- | --- | --- | --- | --- | --- | --- | --- | --- | --- | --- |
| 1.1 Potential for confounding of the effect of intervention? | PY | PY | PY | PY | PN | PY | PY | PY | PY | PY |
| 1.2 Was analysis based on splitting participant’s follow up time? | NI | NI | NI | NI | NI | NI | NI | NI | NI | NI |
| 1.3 Were intervention discontinuations likely related to factors are prognostic for outcome? | NI | NI | NI | NI | NI | NI | NI | NI | NI | NI |
| 1.4 Did authors use appropriate analysis ? | NI | NI | NI | NI | NI | NI | NI | NI | NI | NI |
| 1.5 Were confounding domains controlled for measured validly and reliably? | NI | NI | NI | NI | NI | NI | NI | NI | NI | NI |
| 1.6 Did authors control for any post-intervention variables? | N | N | N | N | N | N | N | N | N | N |
| 1.7 Did authors use apt analysis methods that controlled for all confounding domains? | N | N | N | N | N | N | N | N | N | N |
| 1.8 Were confounding domains measured validly and reliably | NI | NI | NI | NI | NI | NI | NI | NI | NI | NI |
| 1. Risk: | Serious | Serious | Serious | Serious | Serious | Serious | Serious | Serious | Serious | Serious |
| 2.1 Was participant selection after intervention? | N | N | N | N | N | N | N | N | N | N |
| 2.3 Were post interv variables that influenced selection associated with interv? | / | / | / | / | / | / | / | / | / | / |
| 2.4 Do start of f/u and interv coincide? | PY | PY | PY | PY | PY | PY | PY | PY | PY | PY |
| 2.5Were adjust techniques used that are likely to correct for bias? | N | N | N | N | N | N | N | N | N | N |
| 2 Risk | Low | Low | Low | Low | Low | Low | Low | Low | Low | Low |
| 3.1 Interv groups clearly defined? | Y | Y | Y | Y | Y | Y | Y | Y | Y | Y |
| 3.2 Was info recorded at start? | Y | Y | Y | Y | Y | Y | Y | Y | Y | Y |
| 3.3 Could interv classification have been affected by outcome knowledge? | N | N | N | N | N | N | N | N | N | N |
| 3 Risk | Low | Low | Low | Low | Low | Low | Low | Low | Low | Low |
| 4. Deviations from intended interv beyond expected? | NI | NI | NI | NI | NI | NI | NI | NI | NI | NI |
| 4.2 Were these unbalanced b/w groups? | N/NI | N/NI | N/NI | N/NI | N/NI | N/NI | N/NI | N/NI | N/NI | N/NI |
| 4.3 Were co-interventions balanced? | N/NI | N/NI | N/NI | N/NI | N/NI | N/NI | N/NI | N/NI | N/NI | N/NI |
| 4.4 Was interv implemented successfully for most? | NI | NI | NI | NI | NI | NI | NI | NI | NI | NI |
| 4.5 Did participants adhere? | NI | NI | NI | NI | NI | NI | NI | NI | NI | NI |
| 4.6 Apt anaylsis used to estimate effect? | NI | NI | NI | NI | NI | NI | NI | NI | NI | NI |
| 4 Risk | NI | NI | NI | NI | NI | NI | NI | NI | NI | NI |
| 5.1 Were outcome data available for (nearly) all? | N | N | N | N | N | N | Y | Y | Y | Y |
| 5.2 Were participants excluded due to missing data needed for intervention status? | PN | PN | PN | PN | PN | PN | PN | PN | PN | PN |
| 5.3 Were participants excluded due to missing data for analysis | N | NI | NI | NI | NI | NI | N | N | N | N |
| 5.4 Are proportion of participants and reasons similar? | NA | NA | NA | NA | NA | NA | NA | NA | NA | NA |
| 5.5 Evidence that results were robust to missing data | NI | NI | NI | NI | NI | NI | NI | NI | NI | NI |
| 5 Risk | NI | NI | NI | NI | NI | NI | NI | NI | NI | NI |
| 6.1 Could the outcome measure have been influenced by knowledge of the intervention received? | PY | N | N | N | N | N | PY | PY | PY | PY |
| 6.2 Were outcome assessors aware of the intervention received by study participants? | Y | Y | Y | Y | Y | Y | Y | Y | Y | Y |
| 6.3 Were the methods of outcome assessment comparable across intervention groups? | PY | NI | PY | PY | PY | NI | PY | PY | PY | PY |
| 6.4 Were any systematic errors in measurement of the outcome related to intervention received? | NI | NI | NI | NI | NI | NI | NI | NI | NI | NI |
| 6 Risk | Low | Low | Low | Low | Low | Low | Low | Low | Low | Low |
| 7 Is the reported effect estimate likely to be selected on the basis of the results from…. |  |  |  |  |  |  |  |  |  |  |
| 7.1 multiple outcome measurements within the outcome domain? | NI | NI | NI | NI | NI | NI | NI | NI |  | NI |
| 7.2….multiple analyses of the intervention-outcome relationship? | NI | NI | NI | NI | NI | NI | NI | NI |  | NI |
| 7.3…different subgroups? | NI | NI | NI | NI | NI | NI | NI | NI |  | NI |
| 7 Risk | NI | NI | NI | NI | NI | NI | NI | NI |  | NI |

Key: FOAMS = Functional Outcomes Assessment Measure of Swallowing Asp = aspiration, dur = duration, b/w = between, op = operation, d/c discharge, sx = surgery, Pneum = pneumonia, Max = maximum, UES = upper oesophageal sphincter, Interv = intervention, mo = months, fav = favourable

**Overall Risk: Serious.** The study is judged to be at a serious risk of bias in at least one domain, but not at critical risk of bias in any domain.

Case series [69]:

|  | **VFSS findings** | **Independence**  **with oral intake** | **Presence of**  **laryngeal nerve**  **palsy** | **Aspiration pneumonia** |
| --- | --- | --- | --- | --- |
| 1.1 Potential for confounding of the effect of intervention? | Y | Y | Y | Y |
| 1.2 Was analysis based on splitting participant’s follow up time? | N | N | N | N |
| 1.3 Were intervention discontinuations likely related to factors are prognostic for outcome? | NI | NI | NI | NI |
| 1.4 Did authors use appropriate analysis ? | NI | NI | NI | NI |
| 1.5 Were confounding domains controlled for measured validly and reliably? | N | N | N | N |
| 1.6 Did authors control for any post-intervention variables? | NI | NI | NI | NI |
| 1.7 Did authors use apt analysis methods that controlled for all confounding domains? | N | N | N | N |
| 1.8 Were confounding domains measured validly and reliably | N | N | N | N |
| 1. Risk: | Critical | Critical | Critical | Critical |
| 2.1 Was participant selection after intervention? | N | N | N | N |
| 2.3 Were post interv variables that influenced selection associated with interv? | N | N | N | N |
| 2.4 Do start of f/u and interv coincide? | PY | PY | PY | PY |
| 2.5Were adjust techniques used that are likely to correct for bias? | - | - | - | - |
| 2 Risk | Low | Low | Low | Low |
| 3.1 Interv groups clearly defined? | N/A | N/A | N/A | N/A |
| 3.2 Was info recorded at start? | Y | Y | Y | Y |
| 3.3 Could interv classification have been affected by outcome knowledge? | PY | N | N | N |
| 3 Risk | Low | Low | Low | Low |
| 4. Deviations from intended interv beyond expected? | NI | NI | NI | NI |
| 4.2 Were these unbalanced b/w groups? | - | - | - | - |
| 4.3 Were co-interventions balanced? | NI | NI | NI | NI |
| 4.4 Was interv implemented successfully for most? | NI | NI | NI | NI |
| 4.5 Did participants adhere? | NI | NI | NI | NI |
| 4.6 Apt anaylsis used to estimate effect? | NI | NI | NI | NI |
| 4 Risk | NI | NI | NI | NI |
| 5.1 Were outcome data available for (nearly) all? | N | Y | Y | N |
| 5.2 Were participants excluded due to missing data needed for intervention status? | N | N | N | NI |
| 5.3 Were participants excluded due to missing data for analysis | N | N | N | NI |
| 5.4 Are proportion of participants and reasons similar? | - | - | - | - |
| 5.5 Evidence that results were robust to missing data | _ | _ | _ | _ |
| 5 Risk | Moderate | Low | Low | Low |
| 6.1 Could the outcome measure have been influenced by knowledge of the intervention received? | Y | N | N | N |
| 6.2 Were outcome assessors aware of the intervention received by study participants? | PY | PY | PY | PY |
| 6.3 Were the methods of outcome assessment comparable across intervention groups? | Y | Y | Y | Y |
| 6.4 Were any systematic errors in measurement of the outcome related to intervention received? | NI | NI | NI | NI |
| 6 Risk | Moderate | Low/NI | Low/NI | Low/NI |
| 7 Is the reported effect estimate likely to be selected on the basis of the results from…. |  |  |  |  |
| 7.1 multiple outcome measurements within the outcome domain? | NI | NI | NI | NI |
| 7.2….multiple analyses of the intervention-outcome relationship? | NI | NI | NI | NI |
| 7.3…different subgroups? | NI | NI | NI | NI |
| 7 Risk | NI | NI | NI | NI |

**Overall Risk: Critical**. The study is judged to be at a critical risk of bias in at least one domain.

Retrospective Case-control study [70]

|  | **Start of Oral Intake** | **Length of Oral Intake rehabilitation** | **Length of postoperative stay** |
| --- | --- | --- | --- |
| 1.1 Potential for confounding of the effect of intervention? | Y | Y | Y |
| 1.2 Was analysis based on splitting participant’s follow up time? | N | N | N |
| 1.3 Were intervention discontinuations likely related to factors are prognostic for outcome? | NI | NI | NI |
| 1.4 Did authors use appropriate analysis ? | Y | Y | Y |
| 1.5 Were confounding domains controlled for measured validly and reliably? | N | N | N |
| 1.6 Did authors control for any post-intervention variables? | NI | NI | NI |
| 1.7 Did authors use apt analysis methods that controlled for all confounding domains? | Y | Y | Y |
| 1.8 Were confounding domains measured validly and reliably | N | N | N |
| 1. Risk: | Serious | Serious | Serious |
| 2.1 Was participant selection after intervention? | N | N | N |
| 2.3 Were post interv variables that influenced selection associated with interv? | N | N | N |
| 2.4 Do start of f/u and interv coincide? | NI | NI | NI |
| 2.5Were adjust techniques used that are likely to correct for bias? | N | N | N |
| 2 Risk | Low | Low | Low |
| 3.1 Interv groups clearly defined? | Y | Y | Y |
| 3.2 Was info recorded at start? | Y | Y | Y |
| 3.3 Could interv classification have been affected by outcome knowledge? | N | N | N |
| 3 Risk | Low | Low | Low |
| 4. Deviations from intended interv beyond expected? | NI | NI | NI |
| 4.2 Were these unbalanced b/w groups? | NI | NI | NI |
| 4.3 Were co-interventions balanced? | NI | NI | NI |
| 4.4 Was interv implemented successfully for most? | NI | NI | NI |
| 4.5 Did participants adhere? | NI | NI | NI |
| 4.6 Apt anaylsis used to estimate effect? | Y | Y | Y |
| 4 Risk | NI | NI | NI |
| 5.1 Were outcome data available for (nearly) all? | Y | Y | Y |
| 5.2 Were participants excluded due to missing data needed for intervention status? | NI | NI | NI |
| 5.3 Were participants excluded due to missing data for analysis | NI | NI | NI |
| 5.4 Are proportion of participants and reasons similar? | Y | Y | Y |
| 5.5 Evidence that results were robust to missing data | NI | NI | NI |
| 5 Risk | NI | NI | NI |
| 6.1 Could the outcome measure have been influenced by knowledge of the intervention received? | PN | PN | PN |
| 6.2 Were outcome assessors aware of the intervention received by study participants? | PY | PY | PY |
| 6.3 Were the methods of outcome assessment comparable across intervention groups? | Y | Y | Y |
| 6.4 Were any systematic errors in measurement of the outcome related to intervention received? | NI | NI | NI |
| 6 Risk | Low | Low | Low |
| 7 Is the reported effect estimate likely to be selected on the basis of the results from…. |  |  |  |
| 7.1 multiple outcome measurements within the outcome domain? | N | N | N |
| 7.2….multiple analyses of the intervention-outcome relationship? | N | N | N |
| 7.3…different subgroups? | N | N | N |
| 7 Risk | Low | Low | Low |

**Overall Risk: Serious.** The study is judged to be at a serious risk of bias in at least one domain, but not at critical risk of bias in any domain.

**Appendix 7:**

**Downs & Black Methodological Quality Assessment Tool**

| *Reporting* |  | Case Control Study [37] | Case Series [69] | Retrospective Case Series [70] |
| --- | --- | --- | --- | --- |
| 1. *Is the hypothesis/aim/objective of the study clearly described* | yes 1 no 0 | 1 | 0 | 1 |
| 2. *Are the main outcomes to be measured clearly described in the Introduction or Methods section?* | yes 1 no 0 | 1 | 0 | 1 |
| 3. *Are the characteristics of the patients included in the study clearly described ?* | yes 1 no 0 | 1 | 0 | 1 |
| 4. *Are the interventions of interest clearly described?* | yes 1 no 0 | 1 | 0 | 1 |
| 5. *Are the distributions of principal confounders in each group of subjects to be compared clearly described?* | yes 2 partially 1 no 0 | 1 | 0 | 1 |
| 6. *Are the main findings of the study clearly described?* | yes 1 no 0 | 1 | 0 | 1 |
| 7. *Does the study provide estimates of the random variability in the data for the main outcomes?* | yes 1 no 0 | 0 | 0 | 1 |
| 8. *Have all important adverse events that may be a consequence of the intervention been reported?* | yes 1 no 0 | 0 | 0 | 0 |
| 9. *Have the characteristics of patients lost to follow-up been described?* | yes 1 no 0 | 1 | 0 | 0 |
| *10. Have actual probability values been reported(e.g. 0.035 rather than <0.05) for the main outcomes except where the probability value is less than 0.001?* | yes 1 no 0 | 1 | 0 | 1 |
| *External validity* |  |  |  |  |
| 11. *Were the subjects asked to participate in the study representative of the entire population from which they were recruited?* | yes 1 no 0 unable to determine 0 | 0 | 0 | 0 |
| 12 *Were those subjects who were prepared to participate representative of the entire population from which they were recruited?* | yes 1 no 0 unable to determine 0 | 0 | 0 | 0 |
| 13. *Were those subjects who were prepared to participate representative of the recruited population?* | yes 1 no 0 unable to determine 0 | 0 | 0 | 1 |
| 14. *Were staff, places, and facilities where patients were treated representative of treatment most received?* | yes 1 no 0 unable to determine 0 | 0 | 0 | 0 |
| 15. *Was an attempt made to blind those measuring the main outcomes of the intervention?* | yes 1 no 0 unable to determine 0 | 0 | 0 | 0 |
| 16. *If any of the results of the study were based on “data dredging”, was this made clear?* | yes 1 no 0 unable to determine 0 | 1 | 1 | 1 |
| *17. In trials and cohort studies, do the analyses adjust for different lengths of follow-up of patients, or in case-control studies, is the time period between the intervention and outcome the same for cases and controls ?* | yes 1 no 0 unable to determine 0 | 1 | 0 | 0 |
| *18 Were the statistical tests used to assess the main outcomes appropriate?* | yes 1 no 0 unable to determine 0 | 1 | 0 | 1 |
| *19 Was compliance with the intervention/s reliable?* | yes 1 no 0 unable to determine 0 | 0 | 0 | 0 |
| 20. *Were the main outcome measures used accurate (valid and reliable)?* | yes 1 no 0 unable to determine 0 | 1 | 0 | 1 |
| *Internal validity - confounding (selection bias)* |  |  |  |  |
| 21. *Were the patients in different intervention groups (trials and cohort studies) or were the cases and controls (case-control studies) recruited from the same population?* | yes 1 no 0 unable to determine 0 | 1 | 0 | 1 |
| 22. *Were study subjects in different intervention groups (trials and cohort studies) or were the cases and controls (case-control studies) recruited over the same period of time?* | yes 1 no 0 unable to determine 0 | 0 | 0 | 1 |
| 23. *Were study subjects randomised to intervention groups?* | yes 1 no 0 unable to determine 0 | 0 | 0 | 0 |
| 24. *Was the randomised intervention assignment concealed from both patients and health care staff until recruitment was complete and irrevocable?* | yes 1 no 0 unable to determine 0 | 0 | 0 | 0 |
| 25. *Was there adequate adjustment for confounding in the analyses from which the main findings were drawn?* | yes 1 no 0 unable to determine 0 | 0 | 0 | 1 |
| 26. *Were losses of patients to follow-up taken into account?* | yes 1 no 0 unable to determine 0 | 0 | 0 | 0 |
| *Power* |  |  |  |  |
| 27. *Did the study have suffcient power to detect a clinically important effect where the probability value for a difference being due to chance is less than 5%?* Sample sizes have been calculated to detect a difference of x% and y%. | Yes = 1 No = 0 Unable to determine = 0  * Modified as per Trac MH, McArthur E, Jandoc R, et al. Macrolide antibiotics and the risk of ventricular arrhythmia in older adults. CMAJ 2016. DOI:10.1503/cmaj.150901  See Appendix 12 | 0 | 0 | 0 |
| TOTAL Scores |  | 13 | 1 | 15 |

**Appendices 8:**

**TIDieR Checklist**

Case Control Study [37]

| Brief Name: Item 1 | Provide the name or phrase that describes the intervention | Summary Score |
| --- | --- | --- |
|  | Perioperative swallowing rehabilitation (SR) in patients who underwent radical esophagectomy. | 2 |
| Why: Item 2 | Describe any rationale, theory, or goal of the elements essential to the intervention |  |
|  | Previous reports on prophylactic swallowing exercises for patients with head and neck cancer treated with chemoradiation therapy [13, 14],  Many patients still suffer from oropharyngeal  dysphagia following esophagectomy even  without anastomotic obstruction [6].  Videofluoroscopic examinations reveal that reduced  hyoid bone excursion and the UES opening after radical esophagectomy are associated with altered swallowing and a risk of aspiration in the early postoperative period [7, 8].  These abnormalities in oropharyngeal swallowing function after transthoracic esophagectomy are generally associated with reduced laryngeal movement, caused by the surgical reconstruction procedure in the neck, with resulting adhesion  formation, scarring, and inflammation, rather than by inadvertent neurological damage, suggesting a possible positive effect of swallowing rehabilitation (SR) [7–10]. SR, in the form of muscle strengthening programs, has been shown to improve swallowing function in healthy subjects, as well as in neurologically impaired subjects [11,12]. The preventative and therapeutic effects of swallowing  exercises for patients with head and neck cancer undergoing chemoradiation therapy have been well demonstrated [13,14]. However, there have been no reports on SR in the perioperative management of patients undergoing esophagectomy.  The purpose of this study was to assess the preventative and therapeutic effects of perioperative SR in patients undergoing radical esophagectomy | 2 |
| What: Item 3 | **Materials: Describe any physical or informational materials used in the intervention, including those provided to participants**  **or used in intervention delivery or in training of intervention providers. Provide information on where the materials can be**  **accessed (such as online appendix, URL)** |  |
|  | Approximately 2–4 weeks prior to their operation,  each patient was given verbal and written instructions about the preoperative program for esophagectomy, which consisted of smoking cessation, respiratory rehabilitation, and oral care with dental brushing. And presumably about the swallow rehab exercises when this started. Exercises explained in Table 1 of article | 1 |
| What: Item 4 | **Procedures: Describe each of the procedures, activities, and/or processes used in the intervention, including any enabling**  **or support activities.**  **(including referral, screening, case finding, assessment, education, treatment sessions, telephone calls)** |  |
|  | Only the exercises are provided (as laid out above and in table 1 of article) | 1 |
| Who provided: Item 5 | **For each category of intervention provider (such as psychologist, nursing assistant), describe their expertise, background,**  **and any specific training given**  Anyone who was involved in providing intervention |  |
|  | Speech pathologists and nurses. The only information provided is: The patients were instructed by the speech pathologist (N.N.) and nurses in the surgical ward to perform the swallowing exercises five times a day at home and continue after admission to the hospital, up until the day before surgery. | 1 |
| How: Item 6 | **Describe the modes of delivery (such as face to face or by some other mechanism, such as internet or telephone) of the**  **Intervention, who initiated the contact, were sessions interactive and whether it was provided individually or in a group** |  |
|  | It is unclear how much face to face intervention patients received while in hospital but it is presumed that it was mostly independently conducted independently by patients | 0 |
| Where: Item 7 | Describe the type(s) of location(s) where the intervention occurred, including any necessary infrastructure or relevant features |  |
|  | Home, and in hospital when patient was admitted prior to and post-surgery | 1 |
| When and How Much: Item 8 | **Describe the number of times the intervention was delivered and over what period of time including the number of sessions,**  **their schedule, and their duration, intensity, or dose. Timing of intervention in relation to relevant events (e.g. how long after diagnosis and before chemo)** |  |
|  | Expected to be carried out x5 per day but adherence was not reported. Preoperative SR with the period (average ± SD) of  23.0 ± 9.2 days,  Postoperative SR and after a period (average ± SD)  of 26 ± 15.0 days | 1 |
| Tailoring: Item 9 | If the intervention was planned to be personalised, titrated or adapted, then describe what, why, when, and how. Did they provide a rationale for tailoring. Description of any decision points and rules used at each point |  |
|  | General program for everyone | 0 |
| Modifications: Item 10* | If the intervention was modified during the course of the study, describe the changes (what, why, when, and how) How did it differ from original. This item refers to modifications that occur at study level, not individual tailoring. |  |
|  | This was not addressed | 0 |
| How well: Item 11 | **How well Planned: If intervention adherence or fidelity was assessed, describe how and by whom, and if any strategies were used to**  **maintain or improve fidelity, describe them. Fidelity = the degree to which an intervention happened in the way the investigators intended it to. This item and item 12 refer beyond how many participants were issued with the intervention but refers to how well the intervention was received (e.g. how many exercises they did, how they did the exercises, how long they did them for etc.)** |  |
|  | This was not addressed | 0 |
| How well: Item 12* | **Actual: If intervention adherence or fidelity was assessed, describe the extent to which the intervention was delivered as**  **planned** |  |
|  | This was not addressed | 0 |
| Total Score |  | 9 |

Case Series [69]

| Brief Name: Item 1 | Provide the name or phrase that describes the intervention | Summary Score |
| --- | --- | --- |
|  | Rehabilitation in swallowing disorders following esophagectomy for thoracic esophageal cancer | 2 |
| Why: Item 2 | Describe any rationale, theory, or goal of the elements essential to the intervention |  |
|  | It is possible that rehabilitation including complementary measures, such as appropriate reclining posture and selection of food consistencies, is effective in postoperative swallowing disorders for thoracic esophageal cancer | 1 |
| What: Item 3 | **Materials: Describe any physical or informational materials used in the intervention, including those provided to participants**  **or used in intervention delivery or in training of intervention providers. Provide information on where the materials can be**  **accessed (such as online appendix, URL)** |  |
|  | No information provided | 0 |
| What: Item 4 | **Procedures: Describe each of the procedures, activities, and/or processes used in the intervention, including any enabling**  **or support activities.**  **(including referral, screening, case finding, assessment, education, treatment sessions, telephone calls)** |  |
|  | See table 2 for list of exercises and purpose of exercises. No further information provided. | **1** |
| Who provided: Item 5 | **For each category of intervention provider (such as psychologist, nursing assistant), describe their expertise, background,**  **and any specific training given**  Anyone who was involved in providing intervention |  |
|  | Not provided | 0 |
| How: Item 6 | **Describe the modes of delivery (such as face to face or by some other mechanism, such as internet or telephone) of the**  **Intervention, who initiated the contact, were sessions interactive and whether it was provided individually or in a group** |  |
|  | Not discussed | 0 |
| Where: Item 7 | Describe the type(s) of location(s) where the intervention occurred, including any necessary infrastructure or relevant features |  |
|  | Hospital with a rehabilitation department for cancer patients | 1 |
| When and How Much: Item 8 | **Describe the number of times the intervention was delivered and over what period of time including the number of sessions,**  **their schedule, and their duration, intensity, or dose. Timing of intervention in relation to relevant events (e.g. how long after diagnosis and before chemo)** |  |
|  | Not mentioned | 0 |
| Tailoring: Item 9 | If the intervention was planned to be personalised, titrated or adapted, then describe what, why, when, and how. Did they provide a rationale for tailoring. Description of any decision points and rules used at each point |  |
|  | Not mentioned | 0 |
| Modifications: Item 10* | If the intervention was modified during the course of the study, describe the changes (what, why, when, and how) How did it differ from original. This item Refers to modifications that occur at study level, not individual tailoring. |  |
|  | Not mentioned | 0 |
| How well: Item 11 | **How well Planned: If intervention adherence or fidelity was assessed, describe how and by whom, and if any strategies were used to**  **maintain or improve fidelity, describe them. Fidelity = the degree to which an intervention happened in the way the investigators intended it to. This item and item 12 refer beyond how many participants were issued with the intervention but refers to how well the intervention was received (e.g. how many exercises they did, how they did the exercises, how long they did them for etc.)** |  |
|  | Not mentioned | 0 |
| How well: Item 12* | **Actual: If intervention adherence or fidelity was assessed, describe the extent to which the intervention was delivered as**  **planned** |  |
|  | Not mentioned | 0 |
| Total Score |  | 5 |

Retrospective Case-control study [70]

| Brief Name: Item 1 | Provide the name or phrase that describes the intervention | Summary Score |
| --- | --- | --- |
|  | Swallpwing intervention led by a speech-language-hearing-therapis (SLHT)t on postoperative dysphagia (after esophagectomy) | 2 |
| Why: Item 2 | Describe any rationale, theory, or goal of the elements essential to the intervention |  |
|  | To confirm the utility of postoperative continuous interventions led by SLHTIndirect training aimed to strengthen muscles related to swallowing and maintain the flexibility of the oral and pharyngeal structures without eating. | 2 |
| What: Item 3 | **Materials: Describe any physical or informational materials used in the intervention, including those provided to participants**  **or used in intervention delivery or in training of intervention providers. Provide information on where the materials can be**  **accessed (such as online appendix, URL)** |  |
|  | Patient education was listed as being a component of indirect training, however access to this material was not provided.The indirect training program consisted of jaw opening, tongue exercises, the Shaker exercise, a thermal-tactile sitmulation and voice therapy. References provided. Not clear how the participants were educated. | 1 |
| What: Item 4 | **Procedures: Describe each of the procedures, activities, and/or processes used in the intervention, including any enabling**  **or support activities.**  **(including referral, screening, case finding, assessment, education, treatment sessions, telephone calls)** |  |
|  | See table 2 for list of exercises and purpose of exercises. No further information provided. | **1** |
| Who provided: Item 5 | **For each category of intervention provider (such as psychologist, nursing assistant), describe their expertise, background,**  **and any specific training given**  Anyone who was involved in providing intervention |  |
|  | The intervention is reported to be SLT led. No further information is provided as regards expertise or specific training. | 1 |
| How: Item 6 | **Describe the modes of delivery (such as face to face or by some other mechanism, such as internet or telephone) of the**  **Intervention, who initiated the contact, were sessions interactive and whether it was provided individually or in a group** |  |
|  | It appears to be individual face to face intervention patients received while in hospital | 1 |
| Where: Item 7 | Describe the type(s) of location(s) where the intervention occurred, including any necessary infrastructure or relevant features |  |
|  | Inpatient post-operative ward in acute hospital Aichi Cancer Center Hospital | 1 |
| When and How Much: Item 8 | **Describe the number of times the intervention was delivered and over what period of time including the number of sessions,**  **their schedule, and their duration, intensity, or dose. Timing of intervention in relation to relevant events (e.g. how long after diagnosis and before chemo)** |  |
|  | No information was provided on the duration, frequency or intensity of indirect or direct swallow training | 0 |
| Tailoring: Item 9 | If the intervention was planned to be personalised, titrated or adapted, then describe what, why, when, and how. Did they provide a rationale for tailoring. Description of any decision points and rules used at each point |  |
|  | While indirect training was general, the direct training with jelly was based on individual patient performance and participants had fluid and food intake increased based on SLT clinical judgement. | 1 |
| Modifications: Item 10* | If the intervention was modified during the course of the study, describe the changes (what, why, when, and how) How did it differ from original. This item Refers to modifications that occur at study level, not individual tailoring. |  |
|  | This was not addressed | 0 |
| How well: Item 11 | **How well Planned: If intervention adherence or fidelity was assessed, describe how and by whom, and if any strategies were used to**  **maintain or improve fidelity, describe them. Fidelity = the degree to which an intervention happened in the way the investigators intended it to. This item and item 12 refer beyond how many participants were issued with the intervention but refers to how well the intervention was received (e.g. how many exercises they did, how they did the exercises, how long they did them for etc.)** |  |
|  | This was not addressed | 0 |
| How well: Item 12* | **Actual: If intervention adherence or fidelity was assessed, describe the extent to which the intervention was delivered as**  **planned** |  |
|  | This was not addressed | 0 |
| Total Score |  | 10 |

**Appendix 9:**

**PRISMA Abstract Checklist**

| **Section and Topic** | **Item #** | **Checklist item** | **Reported (Yes/No)** |
| --- | --- | --- | --- |
| **TITLE** | | |  |
| Title | 1 | Identify the report as a systematic review. | Yes |
| **BACKGROUND** | | |  |
| Objectives | 2 | Provide an explicit statement of the main objective(s) or question(s) the review addresses. | Yes |
| **METHODS** | | |  |
| Eligibility criteria | 3 | Specify the inclusion and exclusion criteria for the review. | Yes |
| Information sources | 4 | Specify the information sources (e.g. databases, registers) used to identify studies and the date when each was last searched. | Yes |
| Risk of bias | 5 | Specify the methods used to assess risk of bias in the included studies. | Yes |
| Synthesis of results | 6 | Specify the methods used to present and synthesise results. | Yes |
| **RESULTS** | | |  |
| Included studies | 7 | Give the total number of included studies and participants and summarise relevant characteristics of studies. | Yes |
| Synthesis of results | 8 | Present results for main outcomes, preferably indicating the number of included studies and participants for each. If meta-analysis was done, report the summary estimate and confidence/credible interval. If comparing groups, indicate the direction of the effect (i.e. which group is favoured). | Yes |
| **DISCUSSION** | | |  |
| Limitations of evidence | 9 | Provide a brief summary of the limitations of the evidence included in the review (e.g. study risk of bias, inconsistency and imprecision). | Yes |
| Interpretation | 10 | Provide a general interpretation of the results and important implications. | Yes |
| **OTHER** | | |  |
| Funding | 11 | Specify the primary source of funding for the review. | N/A |
| Registration | 12 | Provide the register name and registration number. | In Methods |

*From:* Page MJ, McKenzie JE, Bossuyt PM, Boutron I, Hoffmann TC, Mulrow CD, et al. The PRISMA 2020 statement: an updated guideline for reporting systematic reviews. BMJ 2021;372:n71. doi: 10.1136/bmj.n71

**Appendix 10:**

**PRISMA Checklist**

| **Section and Topic** | **Item #** | **Checklist item** | **Location where item is reported** |
| --- | --- | --- | --- |
| **TITLE** | | |  |
| Title | 1 | Identify the report as a systematic review. | Title |
| **ABSTRACT** | | |  |
| Abstract | 2 | See the PRISMA 2020 for Abstracts checklist. | Complete |
| **INTRODUCTION** | | |  |
| Rationale | 3 | Describe the rationale for the review in the context of existing knowledge. | Background paragraphs |
| Objectives | 4 | Provide an explicit statement of the objective(s) or question(s) the review addresses. | Study Aims |
| **METHODS** | | |  |
| Eligibility criteria | 5 | Specify the inclusion and exclusion criteria for the review and how studies were grouped for the syntheses. | Eligibility Criteria: Participants and Interventions Paragraphs |
| Information sources | 6 | Specify all databases, registers, websites, organisations, reference lists and other sources searched or consulted to identify studies. Specify the date when each source was last searched or consulted. | Search Strategy |
| Search strategy | 7 | Present the full search strategies for all databases, registers and websites, including any filters and limits used. | Appendices 1 and 2  Search Strategy |
| Selection process | 8 | Specify the methods used to decide whether a study met the inclusion criteria of the review, including how many reviewers screened each record and each report retrieved, whether they worked independently, and if applicable, details of automation tools used in the process. | Study Selection  Figure 1 |
| Data collection process | 9 | Specify the methods used to collect data from reports, including how many reviewers collected data from each report, whether they worked independently, any processes for obtaining or confirming data from study investigators, and if applicable, details of automation tools used in the process. | Data extraction |
| Data items | 10a | List and define all outcomes for which data were sought. Specify whether all results that were compatible with each outcome domain in each study were sought (e.g. for all measures, time points, analyses), and if not, the methods used to decide which results to collect. | Data analysis |
|  | 10b | List and define all other variables for which data were sought (e.g. participant and intervention characteristics, funding sources). Describe any assumptions made about any missing or unclear information. | Data analysis |
| Study risk of bias assessment | 11 | Specify the methods used to assess risk of bias in the included studies, including details of the tool(s) used, how many reviewers assessed each study and whether they worked independently, and if applicable, details of automation tools used in the process. | Assessment of Methodological Quality |
| Effect measures | 12 | Specify for each outcome the effect measure(s) (e.g. risk ratio, mean difference) used in the synthesis or presentation of results. | N/A |
| Synthesis methods | 13a | Describe the processes used to decide which studies were eligible for each synthesis (e.g. tabulating the study intervention characteristics and comparing against the planned groups for each synthesis (item #5)). | Data Analysis |
|  | 13b | Describe any methods required to prepare the data for presentation or synthesis, such as handling of missing summary statistics, or data conversions. | Data Analysis |
|  | 13c | Describe any methods used to tabulate or visually display results of individual studies and syntheses. | Data Analysis |
|  | 13d | Describe any methods used to synthesize results and provide a rationale for the choice(s). If meta-analysis was performed, describe the model(s), method(s) to identify the presence and extent of statistical heterogeneity, and software package(s) used. | Data Analysis |
|  | 13e | Describe any methods used to explore possible causes of heterogeneity among study results (e.g. subgroup analysis, meta-regression). | N/A |
|  | 13f | Describe any sensitivity analyses conducted to assess robustness of the synthesized results. | N/A |
| Reporting bias assessment | 14 | Describe any methods used to assess risk of bias due to missing results in a synthesis (arising from reporting biases). | Assessment of Methodological Quality |
| Certainty assessment | 15 | Describe any methods used to assess certainty (or confidence) in the body of evidence for an outcome. | Assessment of Methodological Quality |
| **RESULTS** | | |  |
| Study selection | 16a | Describe the results of the search and selection process, from the number of records identified in the search to the number of studies included in the review, ideally using a flow diagram. | Study Selection, Figure 1 |
|  | 16b | Cite studies that might appear to meet the inclusion criteria, but which were excluded, and explain why they were excluded. | Appendix 3 |
| Study characteristics | 17 | Cite each included study and present its characteristics. | Baseline characteristics, Tables 1,2 |
| Risk of bias in studies | 18 | Present assessments of risk of bias for each included study. | Quality Assessment |
| Results of individual studies | 19 | For all outcomes, present, for each study: (a) summary statistics for each group (where appropriate) and (b) an effect estimate and its precision (e.g. confidence/credible interval), ideally using structured tables or plots. | Results |
| Results of syntheses | 20a | For each synthesis, briefly summarise the characteristics and risk of bias among contributing studies. | Baseline Characteristics and Quality Assessment |
|  | 20b | Present results of all statistical syntheses conducted. If meta-analysis was done, present for each the summary estimate and its precision (e.g. confidence/credible interval) and measures of statistical heterogeneity. If comparing groups, describe the direction of the effect. | N/A |
|  | 20c | Present results of all investigations of possible causes of heterogeneity among study results. | Participant Characteristics |
|  | 20d | Present results of all sensitivity analyses conducted to assess the robustness of the synthesized results. | N/A |
| Reporting biases | 21 | Present assessments of risk of bias due to missing results (arising from reporting biases) for each synthesis assessed. | Quality Assessment |
| Certainty of evidence | 22 | Present assessments of certainty (or confidence) in the body of evidence for each outcome assessed. | Discussion - Limitations |
| **DISCUSSION** | | |  |
| Discussion | 23a | Provide a general interpretation of the results in the context of other evidence. | Main Findings |
|  | 23b | Discuss any limitations of the evidence included in the review. | Results - Quality Assessment |
|  | 23c | Discuss any limitations of the review processes used. | Study Limitations |
|  | 23d | Discuss implications of the results for practice, policy, and future research. | Implications for Policy, Practice and Future Research |
| **OTHER INFORMATION** | | |  |
| Registration and protocol | 24a | Provide registration information for the review, including register name and registration number, or state that the review was not registered. | Methods: Registration |
|  | 24b | Indicate where the review protocol can be accessed, or state that a protocol was not prepared. | Methods: Registration |
|  | 24c | Describe and explain any amendments to information provided at registration or in the protocol. | N/A |
| Support | 25 | Describe sources of financial or non-financial support for the review, and the role of the funders or sponsors in the review. | Page 1 |
| Competing interests | 26 | Declare any competing interests of review authors. | Page 1 |
| Availability of data, code and other materials | 27 | Report which of the following are publicly available and where they can be found: template data collection forms; data extracted from included studies; data used for all analyses; analytic code; any other materials used in the review. | Page 1 |

*From:* Page MJ, McKenzie JE, Bossuyt PM, Boutron I, Hoffmann TC, Mulrow CD, et al. The PRISMA 2020 statement: an updated guideline for reporting systematic reviews. BMJ 2021;372:n71. doi: 10.1136/bmj.n71
